# Ursolic acid exerts anti-cancer activity by suppressing vaccinia-related kinase 1-mediated damage repair in lung cancer cells

**DOI:** 10.1038/srep14570

**Published:** 2015-09-28

**Authors:** Seong-Hoon Kim, Hye Guk Ryu, Juhyun Lee, Joon Shin, Amaravadhi Harikishore, Hoe-Youn Jung, Ye Seul Kim, Ha-Na Lyu, Eunji Oh, Nam-In Baek, Kwan-Yong Choi, Ho Sup Yoon, Kyong-Tai Kim

**Affiliations:** 1 Department of Life Sciences, Pohang University of Science and Technology, Pohang 790-784, Republic of Korea; 2 Division of Integrative Biosciences and Biotechnology, Pohang University of Science and Technology, Pohang 790-784, Republic of Korea; 3 School of Biological Sciences, Nanyang Technological University, Singapore 637551; 4 The Graduate School of Biotechnology and Plant Metabolism Research Center, Kyung-Hee University, Suwon 449-701, Republic of Korea; 5 Department of Genetic Engineering, College of Life Sciences, Kyung-Hee University, Suwon 449-701, Republic of Korea

## Abstract

Many mitotic kinases have been targeted for the development of anti-cancer drugs, and inhibitors of these kinases have been expected to perform well for cancer therapy. Efforts focused on selecting good targets and finding specific drugs to target are especially needed, largely due to the increased frequency of anti-cancer drugs used in the treatment of lung cancer. Vaccinia-related kinase 1 (VRK1) is a master regulator in lung adenocarcinoma and is considered a key molecule in the adaptive pathway, which mainly controls cell survival. We found that ursolic acid (UA) inhibits the catalytic activity of VRK1 via direct binding to the catalytic domain of VRK1. UA weakens surveillance mechanisms by blocking 53BP1 foci formation induced by VRK1 in lung cancer cells, and possesses synergistic anti-cancer effects with DNA damaging drugs. Taken together, UA can be a good anti-cancer agent for targeted therapy or combination therapy with DNA damaging drugs for lung cancer patients.

Tumorigenesis involves uncontrolled cell division, growth, and proliferation, all of which are usually caused by malfunction of certain enzymes. Kinase enzymes, in particular, are critically important for cell division, a complex process that depends on the coordinated action of various kinases over a short period of time. Because these mitotic kinases precisely control cell division, alterations in their activity result in abnormal cellular phenotypes, and many researchers interested in the treatment of cancer have focused on their function. Specifically, mitotic kinases such as the cyclin-dependent kinases (CDKs), the polo-like kinases (PLKs), and the Aurora kinases modulate mitotic progression, and defects in these proteins lead to mitotic arrest, and ultimately to cell death^1^. Although many mitotic kinase blockers have been developed, these are unable to kill cancer cells selectively with limited dosing^2^. In most cases, it was found that the amount of drug required to kill cancer cells also kills normal proliferating cells in the bone marrow, colon, and other proliferating tissues.

Despite this hurdle, mitotic kinases are still considered to be good therapeutic targets because of a cancer-specific feature known as “oncogene addiction”. Normal cells adopt a variety of pathways for cell survival and, therefore, cannot be killed by disturbing one particular pathway. Conversely, cancer cells are usually dependent on one particular pathway for survival. Thus, if it was possible to identify, and block, the critical pathway for a specific cancer, the cancer cells could be specifically targeted and killed. Normal cells would be unaffected because they can adapt to utilize another pathway and survive. Notably, the cancer-addicted pathway often includes mitotic kinases, and therefore, the identification of cancer-addicted oncogenes has been extremely important for the development of targeted therapies. In other words, the identification of ‘druggable’ target genes in specific tumors has been a key area of investigation.

The survival rate for lung cancer patients over a 5-year span is lower than that for the majority of other cancer patients, and the development of new therapies to increase long-term survival has been slow^3^,^4^. Thus, the identification of molecular targets related to lung cancer may be a key to improve survival rates. Recent work has focused on vaccinia-related kinase 1 (VRK1) as a possible drug target for lung cancer treatment. Earlier studies corroborated that VRK1 plays an important role in the lung cancer-specific cell cycle network^5^. VRK1 controls cell division during mitosis by phosphorylating histone H3 on Thr3 and Ser10^6^, which is required for chromosome condensation, and also by phosphorylating barrier-to-autointegration factor (BAF) at Thr2, Thr3, and Ser4, it regulates nuclear envelope formation and dismantling^7^,^8^,^9^. Additionally, VRK1 contributes to the G1 to S phase transition by phosphorylating CRE binding protein (CREB)^10^. It phosphorylates several key transcription factors involved in cell division, including c-Jun^11^ and activating transcription factor 2 (ATF2)^12^ and has further been associated with G0 exit and G1 entry^13^. VRK1 is also a well-known DNA damage repair protein that phosphorylates p53 at Thr18, a key residue for binding with the negative regulator and E3 ubiquitin-protein ligase mouse double minute 2 homolog (MDM2)^14^. During the ionizing radiation (IR)-induced DNA damage response (DDR), VRK1 also plays a role in formation of 53BP1 foci^15^, a damage controlling complex. Consequently, these critical roles for VRK1 suggest that it could be an excellent candidate for lung cancer therapy.

Many plants synthesize compounds to protect themselves, and these molecules are often used for the development of drugs and pharmaceutical agents, as well as in food and cosmetics^16^. Natural compounds have been a major focus of efforts to develop drugs for many diverse diseases, particularly as greater than 70% of modern anti-cancer drugs are derived from or structurally related to natural compounds^16^. Therefore, we assayed a natural compound library and selected candidates that inhibit the function of the druggable’ target, VRK1. Among the several compounds that were identified, UA showed specific inhibitory effect against VRK1.

UA is well known to induce autophagy, anti-inflammation^17^,^18^ and apoptosis by suppressing activities of various molecules such as COX2, iNOS, MMP-9, STAT3 and NF-*κ*B^19^,^20^,^21^,^22^,^23^,^24^. Anti-cancer effects of UA have been also reported in a variety of cancers including breast, leukemia, prostate, skin, and liver cancers^21^,^25^,^26^,^27^,^28^. However, target protein of UA and its cellular mechanism remain elusive.

In this study, we studied molecular characteristics of UA as a candidate inhibitor of VRK1 and its molecular basis of inhibition through nuclear magnetic resonance (NMR) spectroscopy and surface plasma resonance (SPR). Finally, we showed that simultaneous treatment of cancer cells with UA and DNA damaging drugs generates synergistic effects and confirms these findings in a mouse xenograft model.

## Results

### Inhibition of VRK1 kinase activity by UA

**Figure 1:**
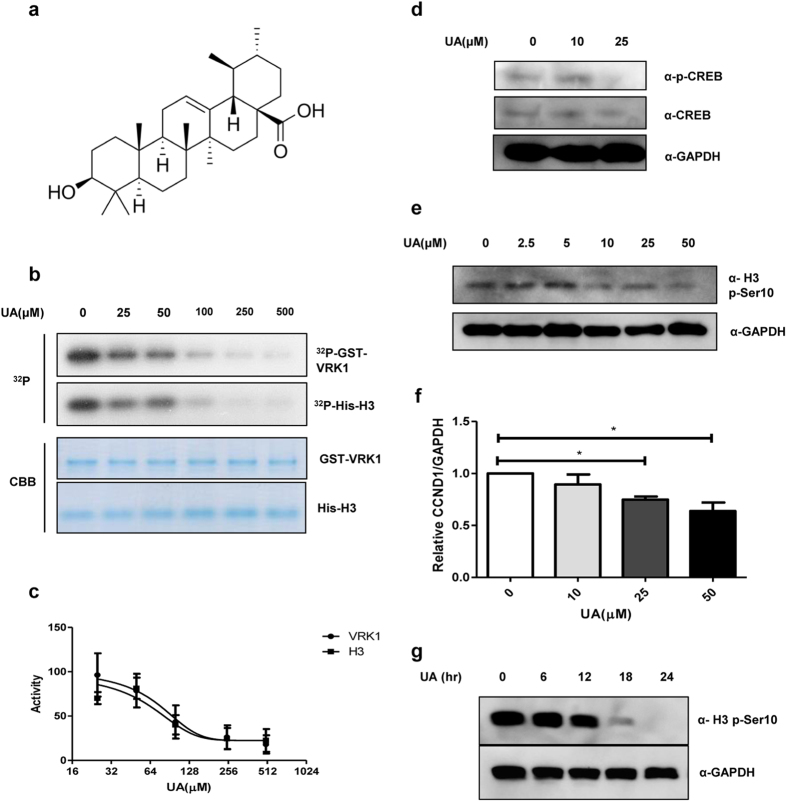
Inhibitory effect of UA on VRK1 kinase activity. (**a**) Chemical structure of UA. (**b**) *in vitro* kinase assay with His-H3 and GST-VRK1 was performed with increasing concentrations of UA (0.0, 25, 50, 100, 250, or 500 μM); GST-VRK1 and His-H3 were then stained with Coomassie blue. (**c**) Quantification of VRK1 auto-phosphorylation and histone H3 phosphorylation shown in panel (**b**). Data represent the mean of three independent experiments ± standard error of means (SEMs). (**d**) Immunoblotting of A549 cell lysates treated with the indicated concentration (0.0, 10, or 25 μM) of UA. (**e**) Immunoblotting of lysates from A549 cells treated with the indicated concentration (0.0, 2.5, 5.0, 10, 25, or 50 μM) of UA. (**f**) Alteration in relative CCND1 mRNA levels after treatment with the indicated concentrations of UA was determined by quantitative real-time PCR; CCND1 mRNA levels were normalized to GAPDH mRNA; error bars indicate the SEM, and asterisk (*) represents *P*-value < 0.05. (**g**) Immunoblotting of A549 cell lysates treated with UA for the indicated times (0, 6, 12, 18, or 24 hr).Immunoblotting was performed using the indicated antibodies, and GAPDH was used as the loading control in all experiments.

We screened a natural compound library to identify VRK1 inhibitors and found several candidate compounds that were able to inhibit the catalytic activity of VRK1. Among these, we selected UA (Fig. 1a) because of its strong inhibitory effect against VRK1, as compared to other compounds. We performed an *in vitro* kinase assay (Fig. 1b) and observed that, in a concentration-dependent manner, UA can inhibit VRK1 auto-phosphorylation, as well as the phosphorylation of histone H3, a known substrate for VRK1. The inhibition curves against both phospho-H3 and phospho-VRK1 were co-related (Fig. 1c), suggesting that UA inhibits VRK1-mediated kinase activity *in vitro*.

VRK1 acts as a mitotic kinase to promote cell cycle progression by phosphorylating various substrates, such as histone H3, CREB, and BAF during each phase of the cell cycle^6^,^7^,^10^. This prompted us to examine UA’s ability to inhibit the catalytic activity of VRK1 in cell culture, as well as *in vitro*. To this end, we employed CREB and histone H3, two representative VRK1 substrates, in lung cancer cells. We showed that UA can inhibit the VRK1-mediated phosphorylation of both CREB and histone H3 *in vivo,* in a concentration-dependent manner (Fig. 1d,e). CREB is a transcription factor that promotes expression of cyclin D1 (CCND1), which is itself a cofactor of the CDK4/6-CCND1 complex at the G1/S transition^10^. To confirm that UA suppresses CREB phosphorylation, we then measured the levels of CCND1 mRNA. Consistent with the decrease in phospho-CREB (p-CREB) levels, CCND1 mRNA levels also decreased in response to UA treatment in a concentration-dependent manner in lung cancer cells (Fig. 1f), suggesting that UA blocks the VRK1 downstream signaling pathway, as well as its enzymatic activity.

We further observed that the UA-mediated inhibition of histone H3 phosphorylation in lung cancer cells was time-dependent (Fig. 1g). Similar to other mitotic kinases, the phosphorylation of substrates by VRK1 oscillates during the progression of the cell cycle. Because inhibitors of these kinases would show effects only at specific cell cycle phases, their inhibitory activity in asynchronous cells is often time-dependent, and accordingly, UA also shows a time-dependent inhibitory effect against VRK1.

### Direct binding of UA to the active site of the VRK1 kinase domain

Because we observed that UA inhibits the kinase activity of VRK1 *in vitro* and in cell culture, we wanted to further to understand the molecular basis of this inhibition. Interestingly, the kinase activity of VRK1 is decreased in a dose-dependent manner by UA treatment *in vitro,* suggesting that other factors are not required (Fig. 1b). This result further implies that UA inhibits the kinase activity of VRK1 *via* direct interactions with this protein.

**Figure 2:**
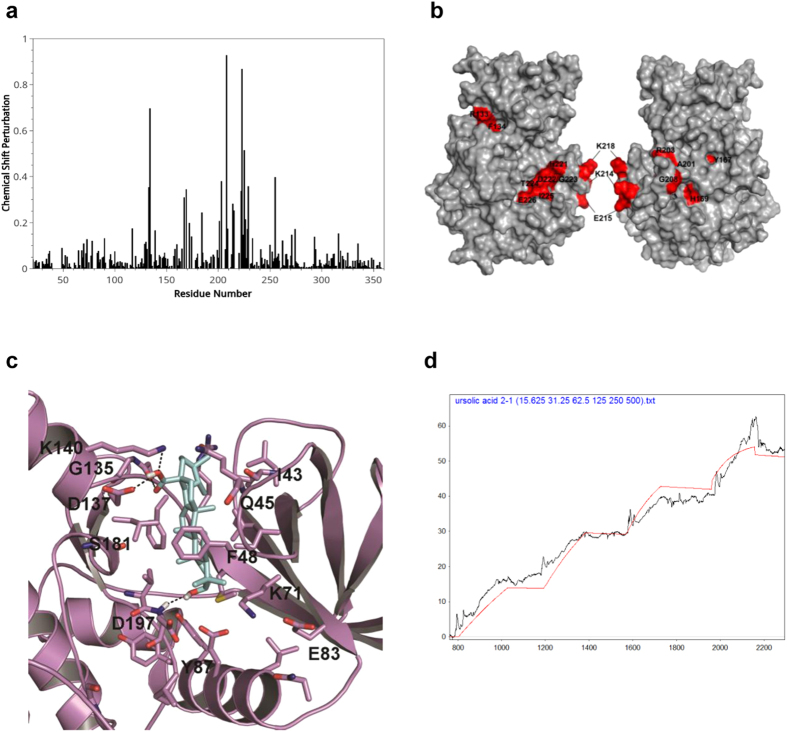
NMR titration assay and *in silico* modeling for interaction of UA with VRK1. (**a**) NMR titration experiments with VRK1 and UA. Spectrum of chemical shift perturbations versus amino acid residues of the VRK1 protein after binding of UA. (**b**) Mapping of chemical shift perturbations on the VRK1 protein. Most of the perturbed residues (shown in red) are located close to the catalytic domain of VRK1. (**c**) Binding pose of UA into active site of VRK1 kinase domain. The carboxyl moiety on UA interacts with main chain carbonyl atoms of G135, side chain atoms of D137 and K140 residues via hydrogen bondings. Likewise the 3-hydroxyl moiety is also engaged in hydrogen bonding interaction with D179 main chain amide atoms. The steroid nucleus makes strong hydrophobic interactions with F48, F134, L184, V69, I51 and K71 residues that outline the VRK1 kinase domain (**d**), SPR detection for the interaction of UA with VRK1. The data were obtained by kinetic titration method with sequential injection of analytes without regeneration steps.

Our previous structural studies provided active site information of VRK1^29^. To determine whether UA can directly bind to VRK1 and to identify the region of VRK1 bound by UA, we conducted NMR titration experiments and *in silico* modeling. Incremental chemical shift perturbations were observed in the amino acid residues of VRK1 upon UA binding (Fig. 2a). These residues were identified on the molecular map of VRK1 (Fig. 2b); they were found to be mainly located in the vicinity of catalytic domain and are involved in ATP binding. Based on *in silico* modeling, UA was predicted to fit into the vicinity of G-loop, catalytic site and C-terminal lobe of VRK1 kinase domain. UA possesses a steroid nucleus and a predominantly hydrophobic moiety, with a hydroxyl and carboxyl substitutions at 3, 4a positions of steroid nucleus. The 4a-carboxyl moiety on UA was suggested to interact with main chain carbonyl atoms of G135 and the side chain carboxyl group and NH atoms of D137 and K140 residues, respectively via hydrogen bonding interactions (Fig. 2c). In addition, the 3-hydroxyl moiety on UA was predicted to be engaged in hydrogen bonding with main chain amide atoms of D197 residue (Fig. 2c). The steroid nucleus was mainly involved in strong hydrophobic interactions with F48 (G-loop); F134, L184 (in the vicinity of DRF motif); V69, I51 and K71 residues that outline the kinase domain. Taken together, these interactions might firmly lock or stabilize the ligand binding and inhibition of VRK1 kinase activity.

Next, in order to measure binding affinity between UA and VRK1, we performed SPR with recombinant His-VRK1 protein and the small molecule UA (Fig. 2d). We observed dissociation constants in the nM range, indicating that these molecules bind one another with a high affinity, similar to the binding observed between other drugs and their targets (Table 1).

### UA induces accumulation of DNA damage by inhibiting VRK1

**Figure 3:**
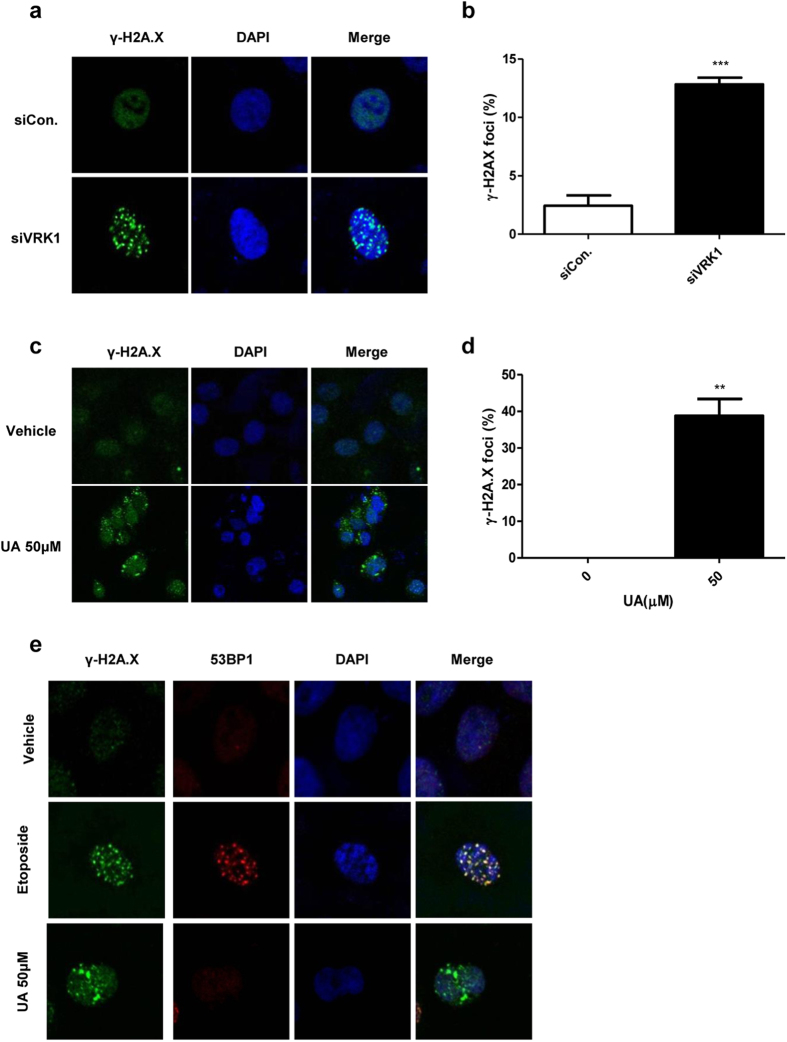
Disruption of DNA damage-induced 53BP1 foci formation by UA. (**a**) Immunocytochemistry of A549 cells transfected with siVRK1 or siCon. (**b**) Quantification of γ-H2A.X positive cells shown in panel (**a**). (**c**) Immunocytochemistry of A549 cells treated with UA or DMSO control. (**d**) Quantification of γ-H2A.X positive cells shown in panel (**c**). (**e**) Immunocytochemistry of A549 cells treated with the indicated compounds. The cells in each group were stained with indicated antibodies and Hoechst, and all images were visualized by confocal laser scanning microscopy. *P*-value was calculated using the Student’s t-test. **P < 0.01, ***P < 0.001.

VRK1 is a kinase involved in the DDR and DNA damage repair. It interacts with, and phosphorylates, p53 at Thr-18, which is critical for increasing p53 protein levels by disrupting the interaction between p53 and the E3 ubiquitin ligase MDM2^14^,^30^,^31^. VRK1 is also known to be involved in 53BP1 foci assembly, by acting as an essential scaffold protein to recruit DNA repair proteins after IR-induced damage^15^. Cells are continually afflicted by endogenous stressors, including the by-products of metabolic pathway and replication stress^32^. Thus, the loss of proteins involved in the surveillance system required for detection of DNA damage could cause such damage to accumulate, even in the absence of exogenous damage. For example, PLK1 is involved in the homologous recombination repair system by phosphorylating Rad51, and the loss of PLK1 causes DNA damage accumulation, without exogenous damage^33^. Further, elevated levels of γ-H2A.X, an early marker for DNA double-stranded breaks (DSBs), have been reported in the testes of VRK1-deficient mice^34^. For these reasons, we predicted that a VRK1 inhibitor might cause accumulation of DNA damage by blocking the function of VRK1 in DDR. In accordance with this, we observed that in lung cancer cells, the siRNA mediated loss of VRK1 also generates γ-H2A.X, suggesting that a lack of VRK1 induces DSBs by interfering with the repair system (Fig. 3a,b). Because UA blocks VRK1 kinase activity, we predicted that treatment with this molecule would also lead to elevated γ-H2A.X, and as expected, we found that UA treatment induces γ-H2A.X in lung cancer cells (Fig. 3c,d), suggesting that UA leads to the accumulation of DNA damage by blocking the kinase activity of VRK1.

Under IR, VRK1 is required to recruit 53BP1 to the site of DSBs, and defective VRK1 cannot assemble these 53BP1 foci during the DDR^15^. Because UA inhibits VRK1 activity, we analyzed UA-induced 53BP1 foci disassembly. Treatment with the DNA damage-inducing drug, etoposide, leads to enhanced 53BP1 foci formation, as well as γ-H2A.X foci generation. The DNA damage was confirmed to be due to etoposide treatment, and the DNA damage repair proteins were recruited to the site of DNA damage, indicating that under these conditions, the repair system is normally activated. In contrast to the etoposide treatment, 53BP1 was not recruited to the DNA damage site after UA treatment (Fig. 3e), suggesting that UA facilitates DNA damage accumulation by preventing the recruitment of repair proteins.

### Synergistic effects of UA with DNA damaging drugs

**Figure 4:**
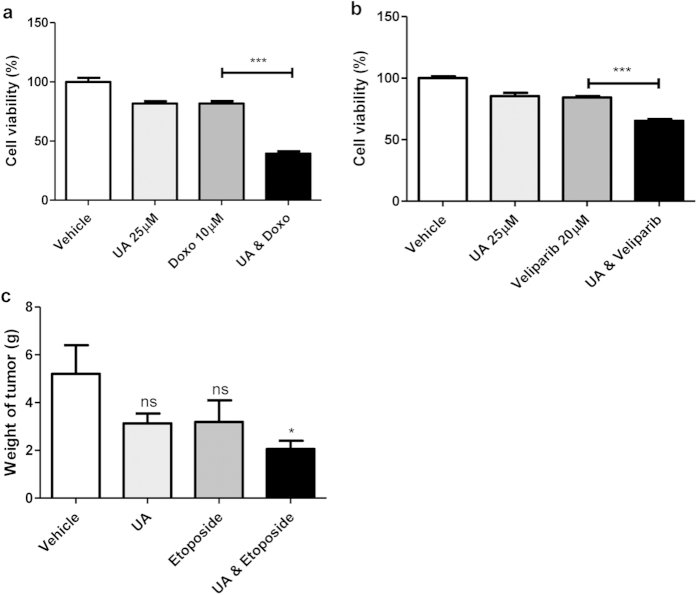
Synergistic effects between UA and the DNA damaging drug. (**a**) Cell viability of A549 cells treated with, or without, doxorubicin and UA. Cell viability was detected with MTT (n = 10). (**b**) Cell viability of A549 cells treated with, or without, veliparib and UA. Cell viability was detected with MTT (n = 10). (**c**) Quantification of *ex vivo* mouse tumor weight after treatment with the indicated compound. *P*-value was calculated using the one-way ANOVA. *P < 0.05, ***P < 0.001.

Cancer cells often depend on specific genes that are essential for cell survival. These target genes could be particularly vulnerable in specific situations, such as in the presence of DNA damage or metabolic stress^35^,^36^. That is, the gene products may act to buffer the effect of stressors/damage; however, if these buffering effects are diminished by mutation or an inhibitor, the cancer cells may be unable to tolerate consistent damage and would eventually undergo cell death^37^. For example, cancer cells with defective pRB or a mutated E2F1 are more sensitive to drugs that elicit DNA damage^38^,^39^,^40^,^41^. It has also been shown that DNA damage inducers render VRK1-deficient lung cancer cells more vulnerable because of the role of VRK1 in DDR^5^,^14^,^15^,^31^,^42^,^43^. Thus, we hypothesized that UA-induced VRK1 inhibition would render lung cancer cells more susceptible to DNA damage. We found that treatment of lung cancer cells with UA, combined with doxorubicin, decreases cell viability more than either compound alone (Fig. 4a). Specificaclly, the EC_50_ for doxorubicin with 24hr incubation was measured to be 56.64 μM, and this EC_50_ was decreased to 6.14 when cells were also treated with 25 μM UA. The combination index, an indicator of synergistic effects between drugs, was found to be 0.66, suggesting that UA-induced VRK1 inhibition has synergistic effects with doxorubicin (Table 2). To investigate whether another drug associated with the inhibition of DNA damage repair genes displays synergistic effects with UA, we incubated UA-treated cells with veliparib, a PARP-1 inhibitor^44^. Although treatment with veliparib alone does not show a cell-killing effect, incubation with veriparib and UA results in a significant decline in cell viability (Fig. 4b).

### Synergistic effects of UA with Doxorubicin in a xenograft mouse model

Finally, to determine whether the synergistic effects between UA and DNA damaging agents occurs *in vivo*, we utilized a xenograft mouse model and performed live imaging to monitor change in tumor size over time. LLC-luciferase cells were injected subcutaneously into the right flank of male BL/6 mice, and the indicated drugs were then intra-peritoneally injected in ventral region of each mouse. We found that after 10 days, although the overall tumor volume was increased in the control group, the volume of tumors in drug treatment group was smaller than tumors in control group. The group treated with UA and doxorubicin, in particular, showed a high synergistic effects (Supplementary Figure S1). To confirm synergistic effects between UA and the DNA damage-inducing drug, etoposide, we injected LLC-luciferase cells into BL/6 mice, and then injected the indicated drugs, as described above. We then measured tumor weight *ex vivo*, and found that, like doxorubicin, etoposide has synergistic effects with UA (Fig. 4c). These results suggest the possibility of developing VRK1 inhibitors as potential anti-cancer therapeutics.

## Discussion

Previous studies have reported that UA, ((1S,2R,4aS,6aR,6aS,6bR,8aR,10S,12aR,14bS)-10-hydroxy-1,2,6a,6b,9,9,12a-heptamethyl-2,3,4,5,6,6a,7,8,8a,10,11,12,13,14b-tetradecahydro-1H-picene-4a-carboxylic acid), a pentacyclic triterpene acid that is widely found in vegetables, inhibits inflammation and upregulates autophagy^17^,^18^. Anti-cancer effects of UA were also reported in a variety of diseases, including breast cancer, leukemia, prostate cancer, lung cancer, melanoma, and endometrial cancer. Specifically, UA was found to inhibit tumor proliferation and tumor cell differentiation, and to induce apoptosis and exert anti-angiogenesis effects^17^,^45^,^46^,^47^,^48^,^49^,^50^,^51^,^52^. The target protein of UA, however, has remained unknown, and therefore, its cellular mechanism has been elusive.

In this study, we identified a target protein for the action of UA and its cell-killing mechanism. We showed that UA binds directly to the catalytic domain of the mitotic kinase, VRK1, and inhibits its kinase activity *in vitro* and in cell culture. By measuring 53BP1 foci formation, we found that UA inhibits the DNA damage defense activity of VRK1 and induces lung cancer cell death. In addition, we showed that co-treatment of lung cancer cells with UA and DNA damaging drugs effectively triggers a more severe cell death than treatment with either UA or drug alone. Finally, we confirmed the synergistic effects between UA and DNA damage-inducing drugs *in vivo* in a xenograft mouse model.

DNA damage triggers severe cellular injury, and in order to quickly recover from this assault, cells require a DNA repair system. Kinases play important roles in DNA repair systems because they have the ability to rapidly propagate the damage signal and to induce immediate reaction. In the DDR, the kinase VRK1 interacts with p53, forming a basal complex, and immediately stabilizes p53 on DNA damage signal^31^. VRK1 phosphorylates p53 at Thr-18, stabilizing the protein and promoting p53-mediated transcription. This leads to elevated expression of genes mediating cell cycle arrest, DNA damage repair, and apoptosis^14^,^30^,^42^,^43^. In addition to p53, c-jun is phosphorylated at Ser-63 and Thr-73 by VRK1, and phosphorylated c-jun turns on DDR-related genes^11^. Furthermore, VRK1 is involved in the recruitment of repair proteins to the sites of DNA damage. In the alternative non-homologous end joining repair process, 53BP1 foci are required to recruit other repair proteins, and VRK1 participates in formation of 53BP1 foci in response to DNA DSBs induced by IR^15^. Because VRK1 functions as an early warning signal through interactions with various other proteins, we expect that UA can be exploited to help elucidate the early response to DNA damage.

VRK1 has been reported to be upregulated in actively dividing cells and in cancer cells, and it is known to be required for G0 exit^13^,^53^,^54^. VRK1 induces cyclin D1 expression by phosphorylating CREB in the G1/S transition, and it is also involved in chromatin compaction by histone H3 in the G2/M transition^6^,^10^. Further, VRK1 deficiency results in infertility due to a progressive loss of spermatogonia in male mice and a defect of folliculogenesis in female mice^55^,^56^,^57^,^58^. VRK1 is one of the key molecules to orchestrate both mitosis and meiosis, and it is an indispensable protein to study cell proliferation and sterility. Therefore, UA, which specifically blocks VRK1, may also help to further understand tumor formation and reproductive sterility.

The nuclear envelope is a structure that segregates the nucleus from cytosol, and its disruption causes cellular catastrophe. Thus, the dynamics of the nuclear envelope determine cell fate. The nuclear envelope is composed of lamin scaffold proteins and other lamin-associated proteins, which help support the envelope structure^59^. A defect in these proteins results in an abnormal nuclear envelope and triggers a devastating disorder known as progeria syndrome^60^. Among the lamin-associated proteins, BAF, in particular, participates in nuclear dynamics. Its unique upstream kinase, VRK1, regulates nuclear envelope breakdown, reassembly, and support of the nuclear structure^7^,^9^,^61^. Defects in VRK1 cause abnormal nuclear envelope structure, likely due to altered BAF function^62^. This suggests that VRK1 is an attractive target in the treatment of cancer, as cancer cells have abnormally fragile nuclear structure and are susceptible to killing under conditions that disturb nuclear envelope dynamics^63^. We have tested this by employing small molecule VRK1 inhibitors that inhibit the VRK1-mediated BAF phosphorylation and consequently prevent nuclear envelope break down or reassembly in cancer cells, suggesting that inhibition of VRK1 renders cancer cells, which contain underlying nuclear envelope defects, more vulnerable^64^,^65^.

Although many small molecules have multiple targets, a VRK1 inhibitor may have more specificity due to the fact that VRK1 has a unique kinase domain, distinct from that of other kinase proteins^29^. Thus, a specific inhibitor of VRK1 may have less cross reactivity with other kinases and fewer undesirable effects. Further, VRK1 proteins also have reduced sensitivity to most inhibitors^66^,^67^; however, current inhibitors of VRK1 have not taken advantage of this circumstance^68^,^69^. Here, we show that UA (K_D_ = 731 nM, EC_50_ = 39 μM) has a stronger binding affinity with VRK1 and a lower EC_50_ against lung cancer cells than luteolin (K_D_ = 5.8 μM, EC_50_ = 59 μM) and other VRK1 inhibitor^69^. Although UA itself might not be sufficient for specific VRK1 inhibition *in vivo*, employing *de novo* structure-based drug design methods or fragment-based approaches at the interacting residues with UA or exploiting structural analogs of UA could facilitate development of novel drugs for the treatment of lung cancer, with minimal side effects.

## Materials and Methods

### Chemicals and Reagents

Analytical grade UA, etoposide and doxorubicin were purchased from Sigma-Aldrich (St. Louis, MO, USA), veliparib was purchased from Selleckchem (Houston, TX, USA), and these compounds were prepared in dimethyl sulfoxide (DMSO) (Sigma-Aldrich) and further diluted in culture medium. The final concentration of DMSO was kept at ≤1 μl/ml. [^32^P-γ] ATP was purchased from Perkin Elmer/NEN (Waltham, MA, USA), and recombinant histone H3 was purchased from Roche Applied Science (Indianapolis, IN, USA). Other recombinant proteins, such as glutathione sulfotransferase (GST), GST-VRK1, and His-BAF were expressed in *Escherichia coli* (BL21) and were purified by affinity chromatography. Glyceraldehyde 3-phosphate dehydrogenase (GAPDH) antibody was purchased from Santa Cruz Biotechnology (Santa Cruz, CA, USA), and histone H3 phospho-Ser10 antibody was purchased from Abcam (Cambridge, UK). Antibodies to phospho-CREB and CREB were obtained from Cell Signaling Technology (Danvers, MA, USA), and those against 53BP1 and γ-H2A.X were purchased from Upstate Biotechnology Inc. (Lake Placid, NY, USA). Hoechst 33342 and CNBr-Sepharose 4B were obtained from Sigma-Aldrich.

### Protein kinase assay and immunoblot

An *in vitro* kinase assay was performed in accordance with methods previously described elsewhere^64^. In brief, the *in vitro* kinase assays were performed in kinase buffer (50 mM MOPS, pH 7.2, 25 mM β-glycerophosphate, 10 mM EGTA, 4 mM EDTA, 50 mM MgCl_2_, 0.5 mM DTT) containing γ-ATP (PerkinElmer), with GST-VRK1 and its substrates, including His-H3 (Fig. 1b). Reactions were carried out for 30 min at 30 °C. Radioactivity incorporation was detected by autoradiography. The quantities of proteins used in kinase assays were measured by using Coomassie blue (Bio-Rad, Hercules, CA, USA). Immunoblot analysis was performed as we previously described elsewhere^64^. For immunoblot analysis, bands were visualized using the Western blot detection kit (Neuronex, Pohang, South Korea).

### Surface plasma resonance

SPR was performed as previously described^64^. His-VRK1 was used as the ligand, and UA was used as the analyte.

### Confocal microscopy

A549 cells transfected with control siRNA (siCon) or siRNA directed against VRK1 (siVRK1) were grown on a microscope slide, and then treated with 50 μM UA or 10 μM etoposide for 12 h. The cells were fixed with paraformaldehyde and permeabilized with 10% fetal bovine serum (FBS; HyClone Laboratories, Logan, UT) in phosphate buffered saline (PBS). Subsequently, cells were stained with indicated antibodies, and the DNA was stained with Hoechst. The cells were then observed using confocal microscopy (Fluoview FV1000; Olympus, Tokyo, Japan).

### Cell Culture

Lewis lung carcinoma cells labeled with a luciferase reporter were obtained from G-one Ahn (Postech, Pohang, Korea). Additionally, A549 cells, which have been previously described, were used in this study^64^. Both cell types were cultured in an RPMI 1640 medium, DMEM high glucose (HyClone) containing 10% FBS (HyClone) and 1% penicillin/streptomycin (Welgene, Daegu, Korea) in a humidified 5% CO_2_ incubator at 37 °C.

### Transfection

Transient transfection was performed by a microporator MP-100 (Invitrogen, Carlsbad, CA) according to manufacturer instructions.

### Quantitative reverse transcription polymerase chain reaction (RT-PCR)

A549 cells were treated with UA at the indicated concentration for 12 h. Total RNA from A549 cells was prepared using the TRI Reagent (Molecular Research Center, Cincinnati, OH, USA) according to the manufacturer instructions, and this was reverse transcribed to generate complementary DNA (cDNA). Quantitative RT-PCR was performed using the SYBR Green PCR mix (Takara Bio Inc., Shiga, Japan) and a real-time detector system (Applied BioSystems, Foster City, CA USA); GAPDH levels were used as a control to normalize transcript levels.

### Plasmids, recombinant protein purification

To generate the BAF and VRK1 expression constructs, full-length BAF and VRK1 were amplified by PCR from HeLa cells, and each DNA fragment was cloned into pEGFP-C1, pDsRed-Monomer-N1, and pProEX (Clontech, Amersham) vectors. To purify recombinant His-VRK1 and His-BAF, pProEX-VRK1 and pProEX-BAF, respectively, were transformed into *E. coli* (BL21), and each protein was purified using Ni-NTA beads (Invitrogen).

### Ligand docking assay

A homology model developed from the X-ray crystal structure of VRK1 was employed to assess the molecular interaction and the binding mode of newly identified VRK1 leads. In the present study, the model structure was energy minimized for 5,000 steps by the CHARM force-field and conjugate gradient method in the Discovery Studio 3.0 suite^29^. The 3-D coordinates of UA were prepared using the Prepare Ligand module and energy minimized for 2,000 steps using the Smart Minimizer algorithm in the Discovery Studio 3.0 suite. The molecular docking program GOLD 5.0 was employed to assess the binding mode of the VRK1 leads. Our previous NMR binding studies have identified the key residues that are perturbed upon ligand binding; these were used to define the active site^29^. Default settings and scoring functions, the GOLD PLP and GOLD scoring function, were employed to score docking interactions and their probable docking mode of binding.

### Cell viability assay

Cells were treated for 24 h with indicated compounds (UA, doxorubicin and veliparib) or with DMSO as a control. Cell viability was assessed using the 3-(4,5-dimethylthiazol-2-yl)-2,5-diphenyltetarazolium bromide (MTT) assay according to the manufacturer’s protocol. The half-maximal effective concentration (EC_50_) was determined using GraphPad Prism (GraphPad, San Diego, CA, USA). The combination index was calculated using the following equation: combination index = (UA) m_50_/(UA) s_50_+ doxorubicin) m_50_/(doxorubicin) s_50_, where (X) m_50_ is the concentration of drug X that will produce a 50% inhibitory effect in the combination; and (X) s_50_ is the concentration of drug X that will produce the same level of effect by itself. Combination index >1 indicates antagonism; combination index <1 indicates synergy; and combination index = 1 indicates an additive effect^70^.

### Statistical Analysis

The Student’s paired two-tailed t-test, ANOVA, and repeated measure ANOVA were used to determine significance. Values of *P* < 0.05, *P* < 0.01, *P* < 0.001 were indicated by *, **, and *** respectively. All error bars shown in this study represent standard deviations.

### Mouse experiments

LLC-luciferase cells (1 × 10^7^ cells/mouse) were injected subcutaneously into the right flank of C57 BL/6 mice (~6–8 weeks old). One week after injection, mice were split into four groups, and administered the following treatments i) DMSO, ii) UA (100 mg/kg) iii) Doxorubicin (2 mg/kg), or iv) UA plus doxorubicin. Each group contains four mice. Each compound was injected four times during a 10 day span. After injection, on the indicated days, luminescence images were acquired by IVIS spectrum (Caliper, Massachusetts, USA) at the Pohang Center of Evaluation of Biomaterials, Pohang Technopark, Pohang, Republic of Korea. In *ex vivo* experiments, etoposide (6 mg/kg) instead of doxorubicin was injected four times over 2 weeks, similar to above, and mice were sacrificed at two weeks after first drug injection, and tumor weight of mice was measured.

### Ethics Statement

Approval of the study protocol was obtained from the Pohang University of Science and Technology Institutional Animal Care and Use Committee (approval number: 2014-03-0002). All animal experiments were carried out according to the provisions of the AnimalWelfare Act, PHS Animal Welfare Policy, and the principles of the NIH Guide for the Care and Use of Laboratory Animals. All mouse lines were maintained at the POSTECH animal facility under institutional guidelines.

## Additional Information

**How to cite this article**: Kim, S.-H. *et al.* Ursolic acid exerts anti-cancer activity by suppressing vaccinia-related kinase 1-mediated damage repair in lung cancer cells. *Sci. Rep.* **5**, 14570; doi: 10.1038/srep14570 (2015).

## Supplementary Material

Supplementary Information

## Figures and Tables

**Table 1:** Dissociation constants of UA on VRK1.

	*ka* (M^−1^s^−1^)	*kd* (s^−1^)	K_D_
Ursolic acid	21.75	1.59 × 10^−5^	731 nM

**Table 2:** The EC_50_ values of Ursolic acid and doxorubicin in A549 cells.

Drugs	EC_50_ (μM)	Notes
Ursolic acid	39	
Doxorubicin	57	
Doxorubicin (in combination with 25 μM Ursolic acid	6	Combination index = 0.7
