# Corrigendum: Ursolic acid exerts anti-cancer activity by suppressing vaccinia-related kinase 1-mediated damage repair in lung cancer cells

**DOI:** 10.1038/srep15864

**Published:** 2015-10-29

**Authors:** Seong-Hoon Kim, Hye Guk Ryu, Juhyun Lee, Joon Shin, Amaravadhi Harikishore, Hoe-Yune Jung, Ye Seul Kim, Ha-Na Lyu, Eunji Oh, Nam-In Baek, Kwan-Yong Choi, Ho Sup Yoon, Kyong-Tai Kim

Scientific Reports 5: Article number: 14570; 10.1038/srep14570published online: 09282015; updated: 10292015

The original version of this Article contained a typographical error in the spelling of the author Hoe-Yune Jung which was incorrectly given as Hoe-Youn Jung.

This has now been corrected in both the PDF and HTML versions of the Article.

