# Supplementary material for: Ursolic acid exerts anti-cancer activity by suppressing vaccinia-related kinase 1-mediated damage repair in lung cancer cells
Source: Sci Rep. 2015 Sep 28;5:14570. doi: 10.1038/srep14570 (PMC4585938; doi:10.1038/srep14570)
Supplement: Supplementary Information [file srep14570-s1.pdf]

Supplementary information for

Ursolic acid exerts anti-cancer activity by suppressing vaccinia related kinase 1-mediated damage repair in lung cancer cells

Seong-Hoon Kim[1], Hye Guk Ryu[1], Juhyun Lee[2], Joon Shin[3], Amaravadhi Harikishore[3], Hoe-Youn Jung[2], Ye Seul Kim[1], Ha-Na Lyu[1], Eunji Oh[4], Nam-In Baek[4], Kwan-Yong Choi[2], Ho Sup Yoon[3,5], and Kyong-Tai Kim[1,2,*]

[1]Department of Life Sciences, [2]Division of Integrative Biosciences and Biotechnology, Pohang University of Science and Technology, Pohang 790-784, Republic of Korea; [3]School of Biological Sciences, Nanyang Technological University, Singapore 637551; [4]The Graduate School of Biotechnology and Plant Metabolism Research Center, [5]Department of Genetic Engineering, College of Life Sciences, Kyung-Hee University, Suwon 449-701, Republic of Korea

***Corresponding author information:**

Kyong Tai Kim, Ph.D., Professor

Division of Integrative Biosciences and Biotechnology, POSTECH

Hyoja Dong, Pohang, Gyeongbuk, Korea 790-784

Tel: 82-54-279-2297

E-mail: ktk@postech.ac.kr

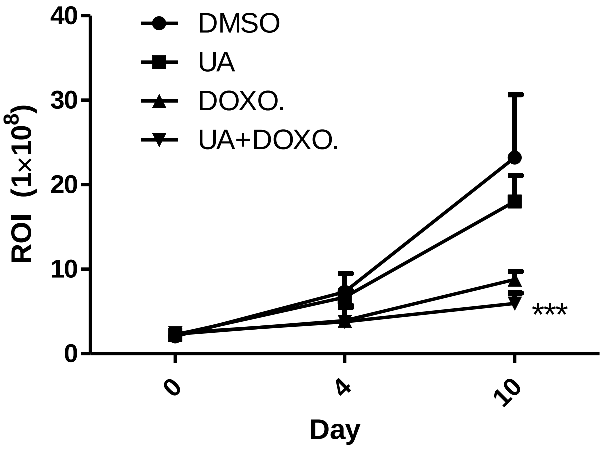

Supplementary Figure 1. Quantification of mouse tumor size after treatment with the indicated compound, recorded using in vivo live imaging. P-value was calculated using repeated measures two-way ANOVA. ***P < 0.001.
